# The influence of HLA genotype on the severity of COVID‐19 infection

**DOI:** 10.1111/tan.14284

**Published:** 2021-05-04

**Authors:** David J. Langton, Stephen C. Bourke, Benedicte A. Lie, Gabrielle Reiff, Shonali Natu, Rebecca Darlay, John Burn, Carlos Echevarria

**Affiliations:** ^1^ ExplantLab, The Biosphere, Newcastle Helix Newcastle‐upon‐Tyne UK; ^2^ Northumbria Healthcare NHS Trust, North Tyneside General Hospital North Shields Tyne and Wear UK; ^3^ Department of Medical Genetics University of Oslo Oslo Norway; ^4^ University Hospital of North Tees Stockton UK; ^5^ Newcastle University Translational and Clinical Research Institute, International Centre for Life (for John Burn) and Population & Health Sciences Institute, Faculty of Medical Sciences, Newcastle University, Newcastle upon Tyne, United Kingdom, for Rebecca Darlay Newcastle‐upon‐Tyne UK; ^6^ Translational and Clinical Research Institute, Newcastle University and Newcastle Hospitals NHS Foundation Trust Newcastle upon Tyne UK

**Keywords:** COVID, COVID‐19, virus, HLA, genetics, latitude

## Abstract

The impact of COVID‐19 varies markedly, not only between individual patients but also between different populations. We hypothesised that differences in HLA genes might influence this variation. Using next generation sequencing, we analysed the class I and class II classical HLA genes of 147 individuals of European descent experiencing variable clinical outcomes following COVID‐19 infection. Forty‐nine of these patients were admitted to hospital with severe respiratory disease. They had no significant pre‐existing comorbidities. We compared the results to those obtained from a group of 69 asymptomatic hospital workers who evidence of COVID exposure based on blood antibody testing. Allele frequencies in both the severe and asymptomatic groups were compared to local and national healthy controls with adjustments made for age and sex. With the inclusion of hospital staff who had reported localised symptoms only (limited to loss of smell/taste, n = 13) or systemic symptoms not requiring hospital treatment (n = 16), we carried out ordinal logistic regression modelling to determine the relative influence of age, BMI, sex and the presence of specific HLA genes on symptomatology. We found a significant difference in the allele frequency of HLA‐DRB1*04:01 in the severe patient compared to the asymptomatic staff group (5.1% vs. 16.7%, *P* = .003 after adjustment for age and sex). There was a significantly lower frequency of the haplotype DQA1*01:01‐DQB1*05:01‐DRB1*01:01 in the asymptomatic group compared to the background population (*P* = .007). Ordinal logistic regression modelling confirmed the significant influence of DRB1*04:01 on the clinical severity of COVID‐19 observed in the cohorts. These alleles are found in greater frequencies in the North Western European population. This regional study provides evidence that HLA genotype influences clinical outcome in COVID‐19 infection. Validation studies must take account of the complex genetic architecture of the immune system across different geographies and ethnicities.

## 1 BACKGROUND

Since it emerged in Wuhan, China in late 2019, COVID‐19 has led to an unprecedented international crisis.^1^ At the time of writing, over 100 million confirmed cases of infection and over three million deaths have been reported to the World Health Organisation.^2^

The impact of COVID‐19 varies markedly, not only between individual patients but also at a national level. At the patient level, it has been shown that males, older patients, those with significant pre‐existing medical conditions, and those with an elevated body mass index (BMI) are at increased risk of poor clinical outcomes following exposure. COVID‐19 also appears to have disproportionately affected certain populations and regions of the world.

Analysis of the impact of COVID‐19 at a population level is complex, requiring consideration of environmental and socioeconomic factors.^3^, ^4^, ^5^ However, clinical variation in COVID‐19 severity and symptomatic presentation may also represent differences in host immunogenetic factors. Through the process of evolution, viruses have exerted selective pressure on humans, meaning that some human populations exhibit marked genetically determined variations in their resistance or susceptibility to different endemic infectious organisms.^6^ It is possible that reported regional variations in the impact of COVID may reflect these variations.^7^, ^8^, ^9^

In this respect, some of the most well described immune features belong to the major histocompatibility complex (MHC). The MHC, located on the short arm of chromosome 6 is the most complex genetic system in the human genome, and includes the HLA genes. The HLA transmembrane proteins encoded by the classical (A, B, C, DR, DQ and DP) HLA genes are principally involved in the antigen presentation, at the cell surface, of small pathogen‐derived peptides to T cells, which triggers an immune response.^10^ Different HLA alleles present different repertoires of peptide fragments from the invading pathogens, potentially influencing the T cell immune response.^11^ HLA gene variants have been implicated in host susceptibility or resistance to diseases such as tuberculosis,^12^ malaria,^9^, ^13^ hepatitis B,^14^, ^15^ dengue,^16^ influenzas,^17^, ^18^ SARS^19^ and MERS.^20^ Bats have evolved highly developed MHCs, and this has been proposed as a reason that they act as a reservoir of coronaviruses, a reservoir from which COVID‐19 may have emerged.^21^, ^22^ Furthermore, the expression of HLA genes is known to be influenced by age,^23^ sex^24^ and obesity,^25^ all identified as key factors in the severity of COVID.

For this study, we investigated whether previously well patients who required admission for treatment of COVID‐19 infection have different HLA alleles compared to asymptomatic controls and the background population.

## 2 METHODS

### 2.1 Study participants and recruitment

As part of an ethically approved research study (‘Do MHC genes play a role in the severity of COVID‐19?’ IRAS project 283409; REC reference: 20/YH/0184) sponsored by North Tees and Hartlepool NHS FT, we compared the classical HLA gene frequencies in two groups: patients admitted with severe COVID infection and hospital staff who remained asymptomatic following exposure to COVID.

### 2.2 Severe COVID patient group

We recruited 49 patients with severe COVID‐19, which was defined as hospitalisation with respiratory failure and a confirmed SARS‐CoV‐2 viral RNA polymerase‐chain‐reaction (PCR) test from nasopharyngeal swabs, from intensive care units and general wards at two teaching hospitals in the North East of England. All patients were of European descent. Respiratory failure was defined as the requirement for oxygen supplementation and/or mechanical ventilation. Only patients with no significant comorbidities were included. Eligible patients (discharged or inpatient) were identified through consultant review of the medical records. Discharged patients were contacted via phone initially. If the patients expressed a wish to participate, they were sent further correspondence including a patient information sheet providing basic information as to the fundamental reasons for the research study. Subsequently, a pack was sent through the post including a saliva collection kit which was mailed back to the research team.

### 2.3 Asymptomatic group

We recruited 69 hospital staff members who had tested positive for COVID infection through routine blood antibody testing. Recruitment was carried out through hospital trust email advertising and review of occupational health records to identify staff members who had tested positive for COVID antibodies on blood screening or had tested positive on swab testing. They were contacted by email or telephone and invited to participate in the research. Blood antibody testing was carried out using the Medicines and Healthcare Regulatory Agency (MHRA) approved Abbott (IL) or Roche tests (Basel, Switzerland) which detect immunoglobulin (Ig) G antibodies to Severe Acute Respiratory Syndrome Coronavirus 2 (SARS‐CoV‐2). For all study participants, patient age, sex, ethnicity and BMI were recorded. Only participants of European descent^26^ were included for this study.

### 2.4 DNA sample collection and processing

A combination of ORAcollect OCR‐100 buccal swabs and Oragene DNA OG‐610 saliva collection kits (both DNA Genotek Inc, Ontario, Canada) were used to collect samples for DNA extraction. DNA was extracted from the swab and saliva samples using the Roche MagnaPure Compact automated platform (Roche Holding AG, Switzerland). DNA was the quantified using the Thermo Fisher Qubit dsDNA BR Assay kit and standardised to 25 ng/μl. HLA genotyping was performed using the One Lambda AllType NGS kits (One Lambda, USA), with the Illumina MiSeq platform (Illumina, USA). Full gene sequencing was carried out for HLA‐A, ‐B, ‐C, ‐DQA1 and ‐DPA1 and partial gene sequencing (omission of exon 1) for HLA‐DRB1, ‐DRB345, ‐DQB1 and ‐DPB1. HLA genotypes were analysed using One Lambda TypeStream Visual 1.3 software (One Lambda, USA).

### 2.5 Control groups: regional and national

Authors of this study are carrying out an ongoing study into the relationship between HLA genotypes and the outcomes of joint replacement surgery. The study involves gene sequencing of patients who have received hip prostheses for end stage degenerative osteoarthritis. The study is being carried out at the same hospital trusts from which the participants of the current study were drawn. 196 patients have undergone full class I and II sequencing and a total of 263 have been sequenced for HLA‐DQ typing. These anonymised data, which included patient age and sex, were used as local control data. These data have previously been compared to a larger national data set comprising 8514 National Blood Service and 1958 Birth Cohort controls and were not shown to significantly deviate.

### 2.6 Statistical analysis

The genotypes for each HLA gene were transformed into dosages of each individual possible allele from within the patient population, where 2 denoted two copies of a given allele, 1 denoted one copy and 0 denoted zero copies. These dosages were then entered as predictor variables in a logistic regression analysis. Three models were tested, the first with the asymptomatic staff versus the severe group, then with asymptomatic staff against background controls, and then with the severe patients as cases against controls. All three models were also tested with sex as an additional covariate and also age plus sex as covariates. Alleles which were found to differ significantly were entered into a binary logistic regression model comparing asymptomatic and severe patients, along with age, sex and BMI.

### 2.7 Ordinal logistic regression

During the recruitment of asymptomatic staff, we received responses from symptomatic individuals who had tested positive for COVID antibodies who expressed their willingness to participate and provide a sample for DNA analysis. The reported symptoms fit into two broad categories:

‘Local symptoms’. These staff members reported symptoms limited to loss of taste and/or smell.

‘Systemic symptoms, no hospital treatment’. These staff members reported systemic upset, general malaise, mild fever and arthralgia.

We incorporated the results from these participants into an ordinal logistic regression model using the following ranking scale:

1. Asymptomatic (n = 69)
2. Local symptoms (n = 13)
3. Systemic symptoms, no hospital treatment (n = 16)
4. Systemic symptoms requiring hospital admission (n = 49)

Age, BMI were used as independent variables, along with sex and presence of specific HLA genes identified in the initial comparison between the severe, asymptomatic and control groups.

Global locus‐wise association for each HLA gene was performed using UNPHASED v 3.0.13. Haplotypes were estimated for DRB1‐DQA1‐DQB1 also in UNPHASED.^27^

#### 2.7.1 Comparison with global data

Previous research has established that latitude relates not only to HLA haplotype frequencies^28^ but also to COVID mortality rates.^29^ In order to place the results of this regional study into context, a parallel analysis was conducted to investigate the relationship between geographic location and HLA gene frequencies in populations from around the world. To do this, we accessed the Allele Frequency Net Database^30^ to record HLA‐DRB1 frequencies in gold standard HLA population studies with at least 100 study participants (n = 151). Logistic regression models were constructed to identify relationships between latitude, longitude and the allele frequencies in populations with these data available (Data S2 and S3). We also examined the influence, if any, of the geographical location of a country and the observed COVID mortality rate. This was carried out using all countries with available socioeconomic data including: median age of population; mean BMI of population and gross domestic product (GDP) per capita (Data S2). We used the same data sources De Larochelambert et al, who carried out an in depth study into the socioeconomic and geographical risk factors associated with increased COVID mortality.^31^

### 2.8 Role of the funding source

The study was funded by Innovate UK. Innovate UK had no role in the study design, the collection, analysis, interpretation of data, the writing of the report, or in the decision to submit the paper for publication.

### 2.9 Results

A total of 147 individuals provided samples. Participant and patient details are shown in Table 1.

**TABLE 1:** Demographics of the study groups

	Group 1 (asymptomatic)	Group 2 (local symptoms)	Group 3 (systemic symptoms)	Group 4 (severe)
Number (total 147)	69	13	16	49
Age (years)	49 (24–69)	43 (27–55)	41 (25–56)	57 (26–77)
M:F	26:43	0:13	4:12	29:20
BMI	27.8 (20.8–42.8)	25.8 (18.4–32.3)	25.8 (20.1–36.1)	30.4 (20.6–49.3)
Median (range) duration of admission	0	0	0	7 (1–56)

#### 2.9.1 HLA gene frequencies in the asymptomatic staff and severe patient groups

The full data set, including calculations performed before and after adjustment for age and sex are shown in Data S1. Locus‐wise association between each HLA locus and severe versus asymptomatic COVID‐19 showed global association for the DRB1 locus (*P* < .008). The alleles which were found to significantly differ between groups are shown in Data S1. The most robust finding was a significant difference in the allele frequency of DRB1*04:01 (split equally into DQA1*03:01‐DQB1*03:02 and DQA1*03:03‐DQB1*03:01) in the severe patient compared to the asymptomatic staff group (5.1% vs. 16.6%, *P* = .003 after adjustment for age and sex). Carrying this allele appeared to protect against severe disease and predispose to an asymptomatic outcome. The frequency of this allele is 11.0% in the study controls and 11.1% in the UK population. DRB1*01:01 (in linkage disequilibrium with DQA1*01:01‐DQB1*05:01) was found at a significantly lower frequency in the asymptomatic group (1.4%) compared to the background populations (9.4% in the study controls and 9.7% in the UK population, *P* = .007). There appeared to be an interaction with sex, with a haplotype frequency in the severe female group of 12.5% compared to 3.4% in the males.

### 2.10 Binary logistic regression

The significant protective effect of HLA‐DRB1*04:01 was retained in the binary regression modelling which also incorporated age, sex and BMI. DRB1*04:01 had approximately the same influence on disease severity as patient sex. Age, however, was the dominant variable (Table 2).

**TABLE 2:** Results of the binary logistic regression model (only asymptomatic hospital staff and severe patient group included)

Variable	Coefficient	Lower bound (95%)	Upper bound (95%)	Standard error	*P* value
Age	0.575	0.280	0.871	0.151	<.001
BMI	0.465	0.187	0.742	0.142	.001
Sex‐M	0.386	0.127	0.645	0.132	.003
DRB1*04:01 carriage	−0.357	−0.630	−0.084	0.139	.010

### 2.11 Ordinal logistic regression

With all confirmed COVID infected cases (n = 147) included in the ordinal regression model, the same variables were found to be significant. Age (coefficient 0.262, *P* = .005), male sex (0.205, *P* = .030) and BMI (0.189, *P* = .045) were associated with more severe symptoms, and carriage of DRB1*04:01 (−0.326, *P* = .001) was associated with less severe symptoms.

#### 2.11.1 Comparison with global data: geographic location, allele frequencies and COVID mortality rates

**FIGURE 1:**
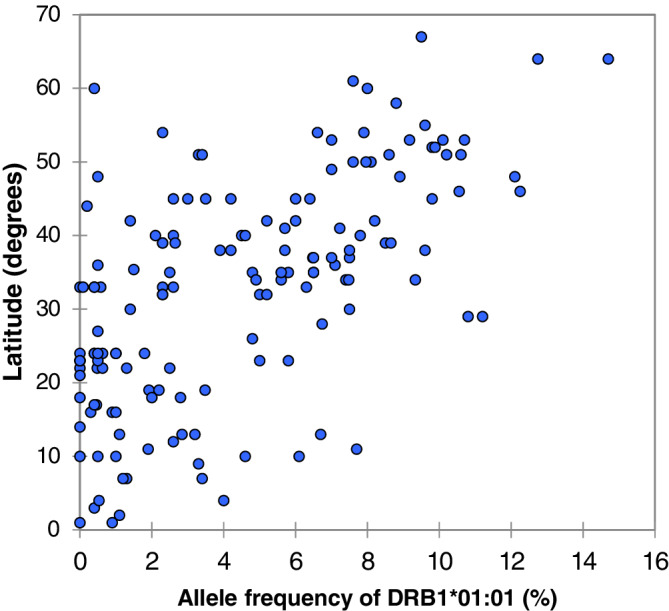
The relationship between latitude and allele frequency of HLA‐DRB1*01:01 in 151 populations from around the world. Spearman rank correlation *r* = .609, *P* ≤ .001

**FIGURE 2:**
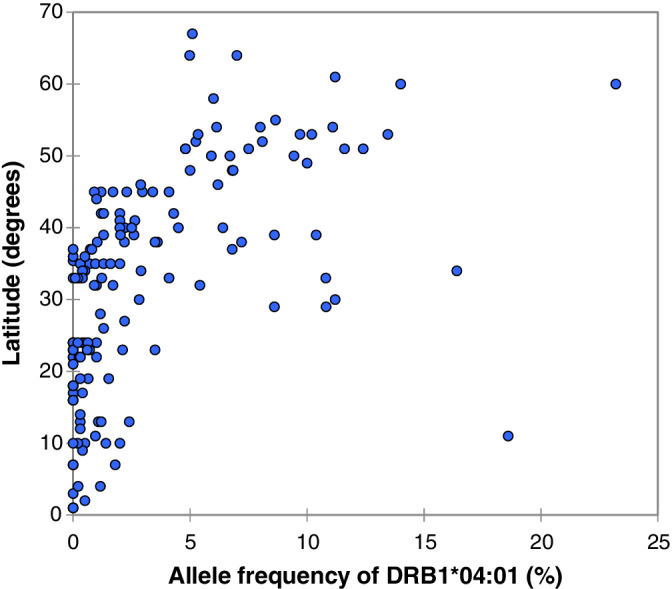
The relationship between latitude and allele frequency of HLA‐DRB1*04:01 in 151 populations from around the world. Spearman rank correlation *r* = .689, *P* ≤ .001

DRB1*04:01 and DRB1*01:01 were found at greater frequencies in populations situated at greater latitudes (Figures 1 and 2) and longitudes closer to 0 degrees. The remaining DRB1 alleles showed weak or non‐significant relationships to latitude (with the exceptions of DRB1*08:01 and DRB1*15:01), being influenced to a greater extent by longitude (Data S2, Table 1). In the global mortality analysis, countries at higher latitudes, with populations with greater mean BMIs were more likely to have greater COVID mortality rates. Longitude and GDP per capita, however, were inversely correlated to mortality rates (Data S2, Table 2). Suitable high resolution HLA data were not available for a sufficient number of countries to repeat the global analysis using DRB1 allelic frequencies as independent variables.

## 3 DISCUSSION

HLA genes are recognised to be important factors in the host response to foreign pathogens. In this regional study, composed of participants of European descent drawn from a relatively small catchment area, we have identified HLA genes which appear to be associated with the severity of COVID‐19 infection.

HLA genes encode for glycoproteins which bind peptides in ‘peptide binding grooves’, present on the cell membrane. HLA class I molecules, which are present on all nucleated cells, present endogenous peptides to cytotoxic CD8+ T cells. HLA class II molecules are expressed on the surface of antigen‐presenting cells (including macrophages, B cells and dendritic cells) and are essential in the presentation of peptides to T‐helper CD4+ cells. The variance of individual amino acids within HLA molecules determine the three dimensional structure of the peptide binding groove. The structure of the binding grooves determines which peptides (which can be foreign or auto antigens) are presented at the cell's surface.^32^

Validated software allows virtual construction of peptide binding grooves encoded by an individual's HLA genotype.^33^ This allows for the calculation of the binding affinity between a particular HLA encoded peptide binding groove and a multitude of naturally occurring peptides. A number of publications have investigated the link between HLA variation and COVID severity using this in silico approach. Some of these studies were combined with epidemiological data^34^, ^35^, ^36^ or results derived from small case control series.^37^ There appears to be no clear consensus in the conclusions drawn so far.

The results of the current study indicate that HLA alleles interact with age, sex and BMI to determine clinical outcomes following COVID exposure. Caution is needed in the interpretation of HLA results; HLA genes are, by their very nature, highly polymorphic. Due to the number of alleles which are under investigation, the risk of identifying a ‘significant’ allele through chance is high^38^ and none of the tests withstood correction for multiple testing. However, the most robust finding appears to be the protection conferred by the presence of DRB1*04:01. Approximately 30% of Europeans carry DRB1*04, an allele with more than 50 subtypes. Of all the DRB1*04 alleles, *04:01 is the most frequent allele occurring with a frequency of 57% as compared to *04:02 which occurs at a frequency of 6.3%.^39^

The various statistical analyses presented in the current article indicate that while DRB1*04:01 may be protective, other DRB1*04 alleles such as DRB1*04:02 and DRB1*04:05 may be associated with an increase in disease severity. On the face of it, this may seem unusual, yet the profound impact of amino acid substitutions and MHC‐restricted T‐cell recognition has long been understood.^40^ For example, DRB1*04:02 and *04:01 molecules differ in the peptide‐binding region by only three amino acids yet they have diametrically opposed clinical outcomes: protection from versus susceptibility to rheumatoid disease.^41^ Certain HLA alleles are superior in providing protection by presenting multiple epitopes for the activation of T cells. The evolutionary trade‐off is that most autoimmune diseases have been associated with the presence of certain HLA class II molecules. This appears to be the case with HLA‐DRB1*04:01, which is associated with rheumatoid disease.^42^ Animal studies comparing 04:01 mice with 04:02 show that, following vaccination, even though both strains of mice are protected from influenza, only *04:01 mice can generate cross‐protective immunity. There is also evidence that MHC peptide loading occurs in different cell compartments between the closely related HLA genes DRB1*04:01 and DRB1*04:02.^17^, ^43^ This fundamental difference between the two molecules may lead to different peptides being loaded and presented despite the same pathogen exposure. Furthermore, in response to superantigens, *04:01 mice have been shown to produce different and varying amounts of cytokines compared to 04:02 strains.^44^

That the strongest predictor of COVID severity was found in the class II HLA‐DR region was not surprising given recently published work.^45^, ^46^ Kachuri et al^3^ conducted a comprehensive study including genome‐wide and transcriptome‐wide association analyses to identify genetic loci associated with IgG antibody response to 16 viruses using serological data from 7924 European ancestry participants in the UK Biobank cohort. Signals in the HLA class II region dominated the antibody response to viruses, with 40 independent loci and 14 independent classical alleles implicated. The strongest associations with seroreactivity were identified within the DRβ1 locus. These results were substantiated by Hammer et al^47^ who carried out a cross‐pathogen, genome‐wide investigation of the role of host genetics in modulating the individual IgG response to common viral antigens. Hammer et al found that individuals carrying DRB1*15:01 were more likely to have detectable levels of anti‐influenza A IgG, whereas the presence of DRB1*01:01 was associated with seronegativity.

Consistent with data from the biobank studies, we found an increased frequency of DRB1*15:01 in both the asymptomatic and severe groups when compared to the background controls. This finding did not achieve significance however in our study. We believe this may have been due to our study protocol, which we designed primarily to identify differences between severe and asymptomatic groups. However, in a study of HLA 99 Italian patients admitted for COVID‐19 infection,^48^ Novelli et al did observe significantly increased frequencies of DRB1*15:01 and DQB1*06:02 (alleles which are recognised to be in strong linkage disequilibrium). The authors noted that their findings conflicted with a larger study by Ellinghaus et al,^37^ who conducted a genome wide association study involving 1980 patients with COVID‐19 and severe disease (defined as respiratory failure) at seven hospitals in Italy and Spain. Ellinghaus et al found no SNP association signals at the HLA complex that met even the significance threshold of suggestive association with either COVID‐19 or disease severity. Ellinghaus et al did, however, identify a 3p21.31 gene cluster as a susceptibility locus and confirmed a potential involvement of the ABO blood‐group system.

Another large GWAS study from the United Kingdom also failed to identify an HLA link.^49^ What could explain the difference between our results and those of the GWAS studies? The difficulties of using GWAS based methods to unpick HLA associations is recognised.^50^ However, in their study, Ellinghaus et al also employed next generation sequencing in a sub set of patients. It could be that the demographics of the study cohorts played a part in the difference in findings. In the Ellinghaus study, the median ages of the patients recruited from seven centres varied from 64 to 69 years with interquartile values ranging from 54 to 79 years. These patients were significantly older and frequently exhibited other medical comorbidities and the hospitals were separated by over 1500 km. In the current study, the median age of the admitted patients was 59 years and all hospitals and the catchment for the control group, were separated by a maximum distance of 80 km. The GENOMICC GWAS identified a signal in the HLA complex which was not confirmed in their validation cohort which was not matched for ethnicity or latitude.^49^ It is noteworthy that a GWAS is challenged where different alleles at the same locus have contrasting effects on phenotype.

The haplotype DQA1*01:01‐DQB1*05:01‐DRB1*01:01 was found at a significantly lower frequency in the asymptomatic group when compared to the background population. The lower frequency of this haplotype in the severe patients suggests that either the response to COVID in these individuals may not be primarily dependent upon IgG antibody production (which would be consistent with biobank data), or that this haplotype is associated with an increased likelihood of developing symptoms. There appeared to be an interaction with patient sex, with the frequency of this haplotype at 12.5% in the severe female group compared to 3.4% in the males. Interestingly, in our ongoing investigation into responses to orthopaedic implants, DRB1*01:01 was significantly associated with a lower probability of T and B cell infiltration into periprosthetic tissues in female patients, and this was one of the factors which prompted the current investigation.

### 3.1 The relationship between geographic location, allelic frequencies and COVID mortality rates

A host of environmental and socioeconomic factors have been shown to influence the course of previous viral outbreaks.^3^, ^51^, ^52^ In light of this, De Larochelambert et al performed an extensive analysis in an attempt to unpick the interaction of health, demographic, environment, and economic parameters on the impact of COVID‐19 in countries across the globe.^31^ They identified several factors associated with COVID mortality rates experienced by a country, which included UV exposure, temperature, humidity, life expectancy and rates of obesity and cancer. The authors substantiated the previously identified positive association between latitude and COVID mortality,^29^ and went on to highlight the inverse relationship between COVID mortality and longitude. We have presented a far simpler statistical analysis in the current article, but the opposing effects of latitude and longitude on COVID mortality held true using a data set which has had another 6 months to accumulate.

Latitude and longitude also correlate to the frequencies of several HLA genotypes.^28^ In the current study, the alleles most strongly related to COVID severity (DRB1*01:01 and DRB1*04:01) were the only alleles to show significant, positive correlations to latitude *and* inverse correlations to longitude. If the results of this study are confirmed, then populations based in North Western Europe have the greatest concentrations alleles associated with both asymptomatic carriage and increased severity.

## SUMMARY

We have provided evidence to show that HLA alleles may interact with patient factors to influence susceptibility to developing severe complications from COVID‐19. HLA alleles associated with asymptomatic carriage of the disease are relatively common in populations of European descent based at higher latitudes.

## CONFLICT OF INTEREST

Based on these results David J. Langton has filed a patent ‘Biomarkers for Covid‐19 Symptom Severity’.

## AUTHOR CONTRIBUTIONS

David J. Langton and Carlos Echevarria designed the research study. DL, Carlos Echevarria, GR and JB collected the data. David J. Langton, Stephen C. Bourke, Benedicte A. Lie, Gabrielle Reiff, Shonali Natu, Rebecca Darlay, John Burn and Carlos Echevarria analysed the data. David J. Langton drafted the manuscript. Stephen C. Bourke, Benedicte A. Lie, Gabrielle Reiff, Shonali Natu, Rebecca Darlay, John Burn and Carlos Echevarria reviewed, modified and approved the final submitted manuscript.

## Supporting information


**DATA S1**: Supporting information

**DATA S2**: Supporting information

**DATA S3**: Supporting information

## Data Availability

The data that support the findings of this study are available from the corresponding author upon reasonable request.
