# Supplementary material for: The influence of HLA genotype on the severity of COVID‐19 infection
Source: HLA. 2021 May 4;98(1):14–22. doi: 10.1111/tan.14284 (PMC8251294; doi:10.1111/tan.14284)
Supplement: Supplementary file 2 — DATA S2: Supporting information [file TAN-98-14-s002.docx]

**Supplementary data 2**

**Relationship of HLA-DR allele frequencies and latitude/longitude**

**Supplementary data 2 table 1:** We accessed the Allele Frequency Net Database to record DRB1 allele frequencies in all available gold standard HLA population studies (with n ≥ 100) and compared them to the latitude and longitude of the corresponding populations (n=151, see supplementary data 3). Longitude and latitude were included as explanatory variables in a multiple regression model to determine their influence on allele frequencies. “Power” refers to the amount of variability explained solely by longitude and latitude. Significant dominant positive correlations to latitude are shown in green; significant correlations to longitude shown in pink. P value refers to the significance of the regression model. The correlation to blood group O is also shown as a comparison.

| **DRB1 allele** | **Power to explain DRB1 frequency** | **Significant variable** | **P value** |
| --- | --- | --- | --- |
| 01:01 | 43% | Latitude +ve > Longitude -ve | <0.001 |
| 03:01 | 51% | Longitude -ve > Latitude +ve | <0.001 |
| 04:01 | 33% | Latitude +ve > Longitude -ve | <0.001 |
| 07:01 | 22% | Longitude -ve | <0.001 |
| 08:01 | 27% | Latitude +ve > Longitude -ve | <0.001 |
| 09:01 | 28% | Longitude +ve | <0.001 |
| 10:01 | 5% | Latitude -ve | 0.009 |
| 11:01 | 16% | Latitude +ve ≈ Longitude -ve | <0.001 |
| 11:04 | 24% | Longitude -ve | <0.001 |
| 12:01 | 19% | Longitude +ve > Latitude +ve | <0.001 |
| 13:01 | 39% | Longitude -ve | <0.001 |
| 13:02 | 4% |  | 0.055 |
| 14:01 | 6% | Longitude +ve | 0.011 |
| 15:01 | 22% | Latitude +ve | <0.001 |
| 15:02 | 21% | Longitude +ve > Latitude -ve | <0.001 |
| **Blood Group O** | **26%** | **Latitude +ve** | **< 0.001** |

**Population location, demographics and COVID mortality**

We took published COVID mortality rates from 152 countries and constructed a regression model including the following explanatory variables: median age of country’s population; mean BMI of country’s population; GDP per capita; latitude and longitude of the country. The significance of the model was p < 0.001 with an R^2^ value of 55%. The inclusion of the percentage of the national population with blood group O did not affect the power of the model (p=0.281).

Data was obtained from: "Global status report on noncommunicable diseases 2014", World Health Organization report published 2018); The UN Human Development Report, 2019 and the “World Economic Outlook - GDP per capita", International Monetary Fund, October 2020.

**Supplementary data 2 table 2**

| Variable | Coefficient | SE | P value | Lower bound (95%) | Upper bound (95%) |
| --- | --- | --- | --- | --- | --- |
| Latitude | 0.231 | 0.088 | **0.010** | 0.057 | 0.405 |
| GDP per capita | -0.175 | 0.072 | **0.016** | -0.318 | -0.033 |
| Median age | 0.173 | 0.101 | 0.089 | -0.027 | 0.373 |
| Mean BMI | 0.501 | 0.077 | **< 0.0001** | 0.350 | 0.652 |
| Longitude | -0.188 | 0.059 | **0.002** | -0.305 | -0.070 |

**Supplementary data 2 figure 1.** Results of the regression modelling for the prediction of COVID mortality across 152 nations.

**United States data**

To further explore the influence of population location and demographics on COVID mortality, we repeated the above regression model using data from each of the 50 states in the United States of America. Mean BMI was replaced by % of the population self-reporting as obese, and GDP per capita was replaced by median household income (Centre for Disease Control data).

Median age of population (p=0.04) and longitude (p=0.001) were the only significant variables to achieve significance in the modelling. These variables both showed inverse correlations to COVID mortality. Stepwise modelling with other socioeconomic parameters (including % of population >65 years of age, % white/black individuals, life expectancy) did not significantly impact on the result.
